# Changes of brain function in patients with type 2 diabetes mellitus measured by different analysis methods: A new coordinate-based meta-analysis of neuroimaging

**DOI:** 10.3389/fneur.2022.923310

**Published:** 2022-08-24

**Authors:** Ze-Yang Li, Teng Ma, Ying Yu, Bo Hu, Yu Han, Hao Xie, Min-Hua Ni, Zhu-Hong Chen, Yang-Ming Zhang, Yu-Xiang Huang, Wen-Hua Li, Wen Wang, Lin-Feng Yan, Guang-Bin Cui

**Affiliations:** ^1^Department of Radiology, Functional and Molecular Imaging Key Lab of Shaanxi Province, Tangdu Hospital, Fourth Military Medical University, Xi'an, China; ^2^Faculty of Medical Technology, Shaanxi University of Chinese Medicine, Xianyang, China; ^3^Battalion of the Second Regiment of Cadets of Basic Medicine, Fourth Military Medical University, Xi'an, China

**Keywords:** functional neuroimaging, type 2 diabetes mellitus, functional magnetic resonance imaging, mild cognitive impairment, coordinated-based meta-analysis

## Abstract

**Objective:**

Neuroimaging meta-analysis identified abnormal neural activity alterations in patients with type 2 diabetes mellitus (T2DM), but there was no consistency or heterogeneity analysis between different brain imaging processing strategies. The aim of this meta-analysis was to determine consistent changes of regional brain functions in T2DM *via* the indicators obtained by using different post-processing methods.

**Methods:**

Since the indicators obtained using varied post-processing methods reflect different neurophysiological and pathological characteristics, we further conducted a coordinate-based meta-analysis (CBMA) of the two categories of neuroimaging literature, which were grouped according to similar data processing methods: one group included regional homogeneity (ReHo), independent component analysis (ICA), and degree centrality (DC) studies, while the other group summarized the literature on amplitude of low-frequency fluctuation (ALFF) and cerebral blood flow (CBF).

**Results:**

The final meta-analysis included 23 eligible trials with 27 data sets. Compared with the healthy control group, when neuroimaging studies were combined with ReHo, ICA, and DC measurements, the brain activity of the right Rolandic operculum, right supramarginal gyrus, and right superior temporal gyrus in T2DM patients decreased significantly. When neuroimaging studies were combined with ALFF and CBF measurements, there was no clear evidence of differences in the brain function between T2DM and HCs.

**Conclusion:**

T2DM patients have a series of spontaneous abnormal brain activities, mainly involving brain regions related to learning, memory, and emotion, which provide early biomarkers for clarifying the mechanism of cognitive impairment and neuropsychiatric disorders in diabetes.

**Systematic review registration:**

https://www.crd.york.ac.uk/prospero/display_record.php?RecordID=247071, PROSPERO [CRD42021247071].

## Introduction

China has the largest diabetes population, with a prevalence rate of 12.8% (1). Type 2 diabetes mellitus (T2DM) accounts for more than 95% of diabetes cases in China, which can lead to a cognitive decline and emotional disorders (1, 2). The report shows that one-quarter of T2DM patients have mild cognitive impairment (MCI) and progress to dementia at a rate of 8.7% per year (3). In addition, T2DM patients may suffer from diabetes-related neuropsychiatric diseases, such as depression, anxiety, and panic, which may develop into depression in future (4, 5). These disorders severely affect the quality of life of T2DM patients (6). Therefore, it is necessary to assess the neural injury of T2DM so as to provide a theoretical basis for effective intervention to delay disease progression.

Functional magnetic resonance imaging (fMRI) is a non-invasive imaging technique for measuring the activity of oxygen-dependent neuronal activity. Together with arterial spin labeling (ASL), fMRI is widely used to study the neuroimaging mechanism of various neuropsychiatric diseases (7–9). Multiple functional and cerebral perfusion indicators such as ALFF, ReHo, ICA, DC, and CBF were used to explore the brain alteration in patients with T2DM, and previous studies showed many functional indicators in the anterior cingulate gyrus, posterior cingulate gyrus, and frontal lobe (10–14). Although the changes in some brain regions were consistent when using different MRI indicators, the studies also found that a large number of brain regions were inconsistent (10–27). This may be due to limited sample size, differences in general clinical patient data, and different methods of measuring the brain activity. Therefore, there is an urgent need for meta-analysis to screen brain dysfunction targets in T2DM patients with more clinical reference value.

Previous meta-analyses including resting-state fMRI (rs-fMRI) and ASL-MRI studies showed robustly reduced resting-state brain activity in the bilateral lingual gyrus, left posterior central gyrus, right inferior temporal gyrus, right cerebellum, right insular lobe, and right posterior cingulate gyrus decreases in T2DM patients, while increased activity in the right precuneus lobe and left superior frontal gyrus (28). This suggests that T2DM had aberrant spontaneous brain activity. Although these findings can provide insights into neuropathological mechanisms of T2DM, previous meta-analyses have some shortcomings. First, the research is based on the activation likelihood estimation (ALE) method (29). As a coordinate-based meta-analysis method, ALE can combine the consistent results of multiple experiments (29, 30), it but lacks sensitivity and heterogeneity analysis (29, 31). This makes it impossible to verify the stability and reproducibility of the meta-analysis results (29–32). In addition, previous studies combined different functional imaging methods for meta-analysis (28, 33), however, the physiological and the pathological significance of these indicators are different when using different neuroimaging methods (11, 14, 34–39).

Thus, we conducted a new meta-analysis using a more reliable and accurate algorithm combining the permutation of subject images (PSI) and Seed-based d Mapping (SDM) software in two groups by different processing methods (14, 38), separately reflecting the relationship between neural activity and the intensity of neural activity in a local brain region (14, 34, 39). This study provides a precise neurobiological mechanism of T2DM causing MCI and can help identify potential early diagnosis and intervention biomarkers.

## Materials and methods

### Protocol and guidance

The study was performed according to the standards of Preferred Reporting Items for Systematic Reviews and Meta-Analysis (PRISMA) (See Supplementary Table S1) and 10 simple rules for neuroimaging meta-analysis (40, 41). The protocol for this neuroimaging meta-analysis was registered on PROSPERO (CRD42021247071) (https://www.crd.york.ac.uk/prospero/).

### Search strategy and study selection

#### Search strategy

The studies were searched in databases including MEDLINE (void), PubMed, Web of Science, Cochrane Library, Elsevier ScienceDirect from 1 January 2007 to 1 December 2021, using the keywords (“Diabetes Mellitus, Type 2” OR “T2DM” OR “Type 2 Diabetes” OR “Diabetes Mellitus, Type II” OR “NIDDM”) AND (“functional magnetic resonance imaging” OR “fMRI” OR “functional neuroimaging” OR “Functional Brain Imaging” OR “ALFF” OR “ReHo” OR “arterial spin labeling” OR “ASL” OR “Cerebral Blood Flow” OR “CBF”) AND (“Cognitive Dysfunction” OR “Mild Cognitive Impairment” OR “MCI” OR “Cognitive Impairment” OR “Cognitive Disorder”) (33, 40). The repetitive and unrelated articles were first excluded, and we further reviewed the references cited in the remaining articles (33). The corresponding author was contacted *via* email if the information was not mentioned in original articles, such as coordinate values and manuscripts not online (41). Our research was restricted to adults and was not limited to the language. In total, two authors (ZY.L. and T.M.) searched the literature and evaluated the quality of the retrieved articles independently (41). If there were any discrepancies, the final decision was made by the third author (LF.Y.) (See Supplementary material Table S1).

#### Inclusion criteria

The studies were included if they met the following conditions: (1) the published article, rather than the abstract, lecture, or letter, (2) studies with comparison made between T2DM and healthy controls, (3) observational studies using a voxel-based analysis to calculate ReHo, ICA, DC, ALFF, or CBF changes in the whole brain, (4) the peak coordinates were clearly reported in stereotactic three-dimensional coordinates (Talairach or Montreal Neurological Institute, MNI) (40), (5) studies from which the *t* value, *z* values, or *p*-values can be extracted, (6) studies using the *t*-test of two independent samples, and (7) those including adults subjects (18–65 years).

#### Exclusion criteria

We excluded studies as follows: (1) systematic review and meta-analysis, (2) the study of the brain structure and task state, (3) T2DM complicated with serious complications (diabetic nephropathy, diabetic retinopathy, diabetic microangiopathy, etc.), (4) studies with no healthy control group, (5) studies with ROI analysis and small-volume correction (40), (6) those with no available coordinates, and (7) the study quality score (QS) <17 (31).

### Meta-analysis of MRI functional alterations

#### Grouping basis

According to different brain activity measurements, we divided the included studies into two groups. The first group reflected the relationship of neural activity in the brain, including ReHo, ICA, and DC (14, 38). ReHo was the most representative one based on the similarity of BOLD signal fluctuation (38), reflecting the uniformity of blood oxygen level-dependent (BOLD) signal in the brain. ICA used the method of blind source separation to separate spatially independent and time series-related functional networks so as to analyze the relationship between networks. DC described the brain network connection strength between a voxel and other voxels in the whole brain, indicating the importance of this voxel as a network node. The second group reflected the intensity of neural activity in the brain, including ALFF and CBF (34, 39). ALFF reflected the average intensity of the low-frequency part of each voxel BOLD signal. CBF indirectly reflected the intensity of neural activity by measuring cerebral blood flow.

#### Voxel-wise meta-analysis

Coordinate-based meta-analysis (CBMA) is a widely used method (31), and we further used a new voxel-based algorithm of the Seed-based d Mapping (SDM) with the permutation of subject images (PSI-SDM version 6.21, https://www.sdmproject.com) (42). The obvious advantage of the new algorithm, which is refer to as PSI, is the use of standard statistical procedures, which avoid the drawbacks of alternative procedures used in current CBMA methods. CBMA uses the statistical alternative procedures, which is not to test whether the effects are invalid but to test whether the reported findings tend to be consistent in some brain regions. A recent study shows that the previous CBMA algorithm has two disadvantages (42). First, it relies on several spatial assumptions, but the data may rarely meet these assumptions, resulting in too liberal results. Second, when brain regions are too scattered, its statistical ability will decline. These adjustments are required for a correct control of the FWER. SDM-PSI estimates the parameters using maximum likelihood techniques to prevent that a single or few studies drive the meta-analysis. This more reliable and accurate algorithm can better control the false-positive rate (42). We divided the brain function indicators into two groups and summarized the abnormal brain activity in type 2 diabetic patients by using PSI-SDM.

Significant peak coordinates were extracted from the literature. Peak coordinates not in the MNI space were converted using coordinate mapping software. Extracting *t* value, *z* value, and *p*-value could be converted into *t* value through https://www.sdmproject.com/utilities/?show=Statistics (42). In total, seven standard steps were preformed according to the guideline of PSI-SDM software: (1) global analysis, (2) pre-processing, (3) mean analysis, (4) threshold analysis, (5) family-wise error (FWE) correction, (6) threshold analysis, and (7) extract and bias test.

The peak coordinates and effect size (e.g., *t* value) of the different brain activity between T2DM patients and healthy controls were extracted from each data set (31). According to the results of the software developer's research, the authors adopted more rigorous methods to control false positives (full width at half-maximum [FWHM] = 20 mm, permutation = 1,000, threshold-free cluster enhancement [TFCE], family-wise error rate [FWER] < 0.05, corrected peak height Z > 1, cluster extent > 10 voxels) (42). It was worth noting that this FWHM kernel was not to smooth the images but to assign effect sizes of propinquity to reported coordinates (31, 43, 44). Finally, BrainNet Viewer (https://www.nitrc.org/projects/bnv/) was used for data visualization (22). In MNI Standard Space Template (Brainmesh ICBM152), volume rendering was used to display significant differences of cortical clusters.

#### Main outcomes

Brain functional alterations between T2DM and healthy controls (HCs) were measured separately. Compared with HCs, the patients with T2DM showed an inconsistent brain activity area related to the cognitive function in the two groups.

#### Comparison analysis

Although the false-positive rate was controlled by using the neuroimaging meta-analysis method based on the permutation of subject images, some valuable brain coordinates were not displayed because the correction is too conservative (42). Based on this, we also used AES-SDM (Anisotropic Effect Size SDM v5.15 https://www.sdmproject.com) to analyze this data set (44). The first cut threshold ([FWHM] = 20 mm, *p*-value < 0.0005 uncorrected, peak height Z > 1, cluster extent > 50 voxels) was set more strictly than the default of the software ([FWHM] = 20 mm, *p*-value < 0.005 uncorrected, peak height Z > 1, cluster extent > 10 voxels) (42, 44). Finally, we showed the results of the two versions.

#### Heterogeneity, publication bias, and evidence quality

After FWE correction based on TFCE (*p* < 0.05), we used PSI-SDM to extract the coordinates of abnormal brain areas and their effect sizes (42). First, the heterogeneity analysis was analyzed using PSI-SDM and I2 tests in Stata/SE 16.0 (Stata Corp LP, College Station, TX, USA). We adopted fixed-effects models (I2 < 50%) or random-effects models (I2 ≥ 50%) to incorporate effect size (45, 46), if there was no significant heterogeneity. Second, funnel plots and Egger tests were performed to evaluate publication bias in our study (47, 48). Third, based on previous high-quality meta-analysis, the quality of the studies was assessed by examining checklists (Supplementary Table S2), which was a specific method of neuroimaging meta-analysis evaluation (31). At the same time, we also used the Joanna Briggs Institute (JBI) critical appraisal checklist (49) to evaluate study quality (Supplementary Table S2). The quality of the articles was independently assessed by two reviewers.

#### Jackknife sensitivity analysis

Jackknife sensitivity was analyzed by AES-SDM (42, 44). After preprocessing, sensitivity analysis was performed by repeating the same analysis in the main interface of AES-SDM (44). One study was excluded at a time to test the stability and repeatability of the meta-analysis (31).

#### Intra-class correlation analysis

The intra-class correlation (ICC) is a descriptive statistic that can be used when quantitative measurements are made on units that are organized into groups (50). It describes how strongly units in the same group resemble each other. In functional connectomics, the ICC is commonly used to quantify its test–retest reliability (50). The ICC was obtained from the relationship between between-group mean square (MSB) and within-group mean square (MSW) by IBM SPSS 20 software.

#### Subgroup and meta-regression analyses

To identify whether data processing methodology influenced the brain functional alterations, subgroup meta-analysis was performed in functional studies using ReHo, ICA, and DC, respectively.

To explore the potential influence of demographic and clinical variables including education, T2DM duration, HbA1c, and the severity of cognitive impairment (Mini-Mental State Examination [MMSE] score and Montreal Cognitive Assessment [MoCA] score) in T2DM brain functional activity, a random-effects general linear meta-regression in AES-SDM was conducted (31, 33). Statistical significance was determined to use a stringent threshold of FWHM = 20 mm, *p* < 0.0005, cluster-corrected, peak height Z > 1, and cluster >10 voxels in meta-regression analysis. Regression analysis excluded findings outside the brain regions detected in the main meta-analysis (33).

## Results

### Eligible studies and sample characteristics

**Figure 1:**
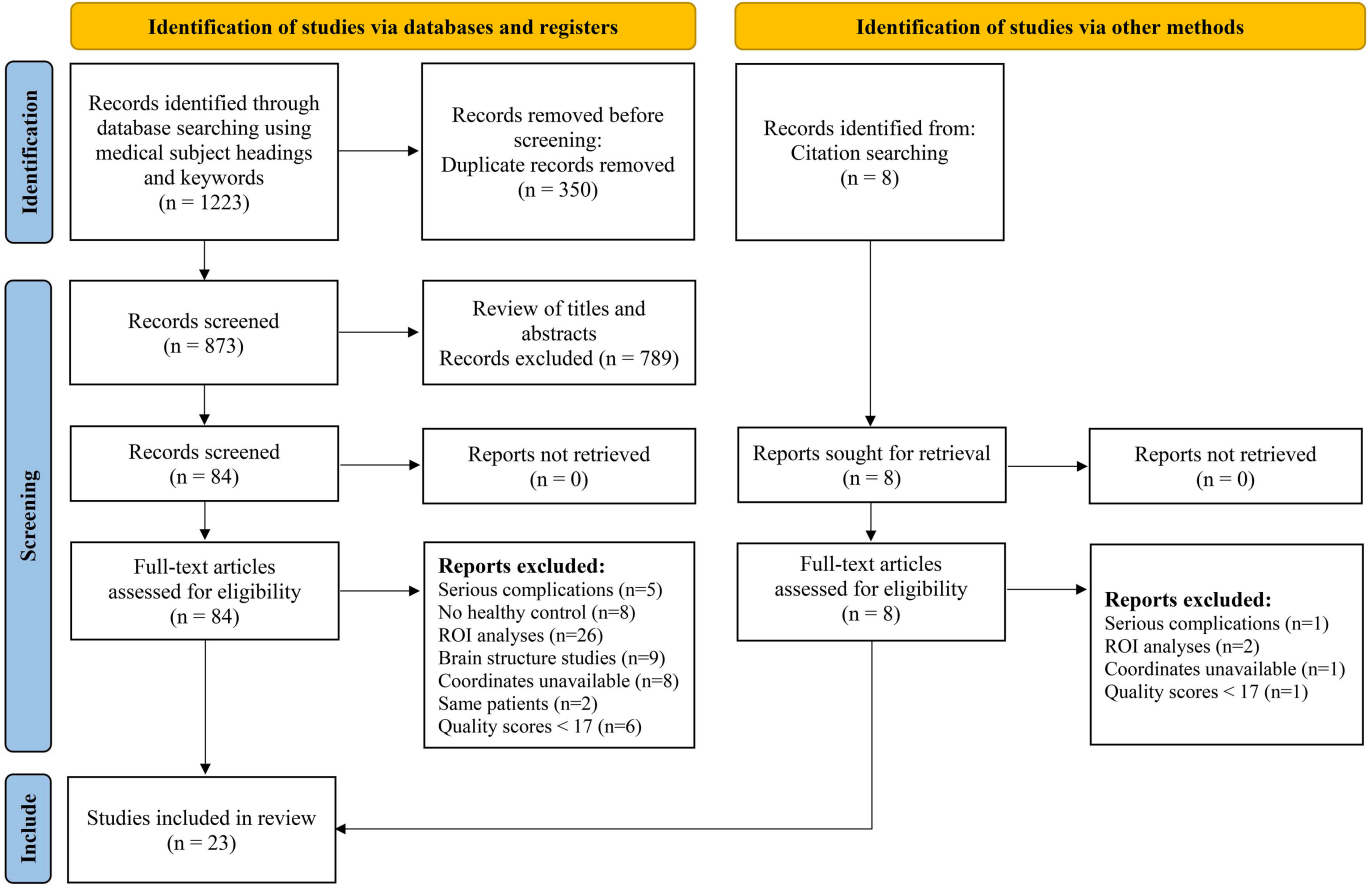
Flowchart to identify the eligible studies for meta-analysis. ROI, region of interest.

We initially identified 1,231 records and eliminated 1,139 by deleting duplicate records and reading titles and abstracts. We scrutinized references in studies that have not been ruled out, seven more references were found that met the criteria for inclusion. We excluded 69 studies after reading the full text because six studies had serious complications (51–56), eight studies had no healthy controls (57–64), 28 studies had ROI analyses only (65–92), nine studies were methods of brain structural analysis (93–101), nine studies did not report coordinates (102–110) (sent to the author for help, no results), the data used in the two studies are the same as the articles included in this study (111, 112), and seven studies had a quality score of less than 17 (113–119). The final meta-analysis included 23 eligible trials with 27 data sets (Figure 1). We divided them into two groups based on different brain imaging processing strategies (11, 14, 34, 38, 39). The first group consisted of 17 data sets, including 553 patients with simple T2DM, and the second group included 11 data sets, with 340 patients. (Detailed general demographic, clinical, and radiographic features are shown in Tables 1–4) On the one hand, we observed an unbalanced sex distribution between T2DM and HCs in the first group ( *x*^2^ = 13.423, *P* < 0.001). Significant differences were observed between T2DM and HCs regarding age (standardized mean difference [SMD] = 0.30, 95% confidence interval [CI] = [0.19, 0.42], Z = 5.04, *P* < 0.00001), there was no difference in the education level (SDM = 0.14, CI = [−0.05, 0.34], Z = 1.42, *P* = 0.16) and MMSE score (SDM = −0.16, CI = [-0.33, 0.02], Z = 1.75, *P* = 0.08) between the T2DM and HC groups. However, there were statistically significant differences in MOCA scores (SDM = −0.91, CI = [−1.42, −0.40], Z = 3.48, *P* = 0.0005). Among them, there was a gender-specific study (123), two education-level studies (121, 123), two MMSEs (16, 17), and six MOCA studies (11, 15, 21, 120–122), which were not analyzed as detailed data on the subjects' indicators were not provided. On the other hand, in the second group, there was no significant difference in age (SDM = 0.12, CI = [−0.03, 0.27], Z = 1.51, *P* = 0.13), education level (SDM = −0.06, CI = [−0.21, 0.10], Z = 0.72, *P* = 0.47), and MMSE score (SDM = −0.16, CI = [−0.40, 0.08], Z = 1.32, *P* = 0.19) between T2DM patients and HC, while there was significant difference in sex ( *x*^2^ = 6.428, *P* < 0.01) and MOCA score (SDM = −1.19, CI=[−1.98, −0.39], Z = 2.93, *P* = 0.003). Among them, four research studies on MOCA have no data (12, 13, 57, 124).

**Table 1:** Demographic, clinical, and cognitive characteristics of study patients and control subjects included in the meta-analysis (group 1).

**Study**	**Sex (male/female)**		**Mean Age (SD)**		**Education years (SD)**		**T2DM duration**		**HbA1c (%) (SD)**		**MMSE (SD)**		**MoCA (SD)**	
							**(SD) in years**							
	**T2DM**	**HC**	**T2DM**	**HC**	**T2DM**	**HC**	**T2DM**	**HC**	**T2DM**	**HC**	**T2DM**	**HC**	**T2DM**	**HC**
Cui et al. (10)	14/15	11/16	58.3 (7.3)	57.8 (5.9)	10.4 (4.0)	10.2 (2.5)	9.3 (3.8)	–	7.9 (1.7)	5.6 (0.4)	28.3 (1.4)	29.0 (1.1)	23.6 (2.9)	27.3 (1.1)
Cui et al. (15)	23/19	14/28	60.4 (7.0)	58.2 (6.3)	9.6 (3.8)	10.2 (2.3)	9.4 (4.7)	–	7.9 (1.6)	5.6 (0.3)	28.3 (1.1)	28.7 (1.2)	–	–
Xia et al. (21)	21/17	17/15	58.6 (8.2)	55.6 (7.1)	9.7 (3.4)	10.4 (2.0)	9.9 (5.7)	–	7.7 (1.7)	5.1 (0.5)	28.8 (1.1)	29.1 (1.2)	–	–
Liu et al. (24)	12/9	10/15	61.7 (5.3)	58.0 (7.9)	9.8 (2.7)	10.1 (2.8)	7.9 (5.5)	–	9.6 (2.6)	–	28.5 (1.5)	27.7 (1.4)	21.8 (1.4)	26.6 (0.8)
Cui et al. (11)	19/21	13/30	60.5 (6.9)	57.6 (6.6)	10.0 (3.4)	10.2 (2.3)	8.9 (5.0)	–	7.8 (1.6)	5.6 (0.3)	28.3 (1.2)	28.7 (1.2)	–	–
Liu et al. (18)	17/8	13/12	52.2 (4.8)	52.1 (3.5)	11.0 (3.1)	11.2 (2.3)	7.7 (5.4)	–	8.5 (1.7)	5.7 (0.5)	27.9 (1.9)	28.2 (1.5)	23.0 (2.9)	24.4 (2.6)
Peng et al. (120)	10/12	12/16	58.8 (7.9)	56.2 (6.9)	10.0 (2.1)	10.4 (2.1)	10.9 (3.4)	–	8.1 (2.2)	5.5 (0.3)	28.9 (0.7)	29.2 (0.6)	–	–
Qu et al. (121)	11/15	10/14	57.6 (9.3)	54.2 (7.8)	–	–	12.1 (5.8)	–	8.9 (1.7)	4.7 (0.6)	27.6 (1.6)	28.3 (1.3)	–	–
Peng et al. (122)	22/26	16/24	58.2 (8.6)	56.3 (6.6)	10.1 (2.9)	10.3 (2.1)	12.5 (–)	–	8.5 (1.9)	5.4 (0.2)	28.9 (0.7)	29.2 (0.6)	–	–
Liu et al. (19)	28/19	25/22	58.7 (6.9)	57.4 (5.4)	10.6 (3.1)	10.8 (2.7)	8.9 (6.6)	–	8.3 (2.1)	5.6 (0.4)	28.3 (1.5)	28.6 (0.8)	23.2 (2.9)	24.5 (2.3)
Yu^a^ et al. (14)	28/5	22/11	53.5 (8.4)	51.0 (5.3)	12.8 (2.4)	12.9 (3.5)	7.1 (5.2)	–	8.1 (1.7)	5.6 (0.3)	28.9 (0.9)	28.5 (1.1)	26.5 (2.1)	26.8 (2.0)
Yu^b^ et al. (14)	28/5	22/11	53.5 (8.4)	51.0 (5.3)	12.8 (2.4)	12.9 (3.5)	7.1 (5.2)	–	8.1 (1.7)	5.6 (0.3)	28.9 (0.9)	28.5 (1.1)	26.5 (2.1)	26.8 (2.0)
Xia et al. (25)	24/20	24/26	58.6 (7.4)	56.3 (6.4)	11.1 (1.7)	11.9 (2.1)	7.6 (6.0)	–	8.1 (2.7)	5.0 (0.4)	28.6 (1.1)	29.1 (1.1)	27.2 (1.4)	27.1 (1.6)
Li et al. (17)	22/12	19/19	49.4 (5.6)	47.4 (6.8)	9.4 (2.4)	10.2 (2.3)	–	–	8.7 (2.2)	–	–	–	26.3 (0.7)	28.0 (0.9)
Liu et al. (123)	26^c^	26^c^	51.9 (10.7)	48.2 (6.7)	–	–	–	–	–	–	26.9 (3.9)	28.7 (1.2)	23.5 (5.6)	26.1 (1.6)
Xiong et al. (26)	10/15	12/15	59.1 (6.2)	59.1 (6.4)	11.6 (3.3)	11.2 (2.6)	5.7 (4.6)	–	7.2 (1.4)	5.3 (0.4)	28.6 (1.0)	28.4 (1.0)	28.1 (0.7)	28.8 (1.1)
Feng et al. (16)	14/6	7/13	36.5 (3.7)	34.1 (4.8)	11.9 (4.6)	14.4 (3.2)	3.8 (0.8)	–	9.8 (2.1)	–	–	–	27.0 (2.8)	28.0 (2.8)

Data are presented as mean (SD), or ratios. T2DM, type 2 diabetes mellitus; HC, healthy control; SD, standard deviation; HbA1c, glycosylated hemoglobin A1c; MMSE, mini-mental state examination; MoCA, montreal cognitive assessment.

a Data set of the indicator of degree centrality (DC).

b Data set of the indicator of mean regional homogeneity (mReHo).

c Sample size.

**Table 2:** Imaging characteristics of the studies included in the meta-analysis (group 1).

**Study**	**Scanner**	**Scan**	**Software**	**Indicator**	**Frequency**	**Smoothing**	**Threshold (method)**	**GMV**	**Quality**
		**duration**			**range (Hz)**	**kernel (mm)**		**correction**	**scores^a^**
Cui et al. (10)	3.0 T	>6 min	SPM8, DPARSF, REST	ReHo	0.01–0.08	4	*P* <0.01 (AlphaSim corrected)	Yes	17
Cui et al. (15)	3.0 T	8 min	SPM8, GIFT	ICA	<0.1	4	*P* <0.05 (AlphaSim corrected)	Yes	20
Xia et al. (21)	3.0 T	8 min 6 s	SPM8, DPARSF, REST, GIFT	ICA	0.01–0.08	4	*P* <0.05 (FDR corrected)	Yes	18
Liu et al. (24)	3.0 T	8 min	SPM8, DPARSF, REST	ReHo	0.01–0.08	4	*P* <0.05 (AlphaSim corrected)	No	17
Cui et al. (11)	3.0 T	6 min	SPM8, DPARSF, AFNI	DC	0.01–0.1	4	P <0.05 (AlphaSim corrected)	No	17
Liu et al. (18)	3.0 T	8 min 8 s	SPM8, DPARSF, REST	ReHo	0.01–0.08	4	*P* <0.01 (AlphaSim corrected)	Yes	19
Peng et al. (120)	3.0 T	8 min	SPM8, DPARSF, REST	ReHo	0.01–0.08	4	*P* <0.05 (AlphaSim corrected)	Yes	20
Qu et al. (121)	3.0 T	8 min	SPM8, DPARSF, REST	ReHo	0.01–0.08	6	*P* <0.05 (FDR corrected)	Yes	17
Peng et al. (122)	3.0 T	8 min	SPM8, DPARSF, REST	ReHo	0.01–0.08	6	*P* <0.05 (FWE corrected)	Yes	17
Liu et al. (19)	3.0 T	8 min	SPM8, DPARSF, REST	DC	0.01–0.08	4	*P* <0.001 (AlphaSim corrected)	Yes	19
Yu^b^ et al. (14)	3.0 T	>6 min	SPM8, DPABI, REST	DC	0.01–0.1	8	*P* <0.05 (GRF corrected)	Yes	20
Yu^c^ et al. (14)	3.0 T	>6 min	SPM8, DPABI, REST	mReHo	0.01–0.1	8	*P* <0.05 (GRF corrected)	Yes	20
Xia et al. (25)	3.0 T	>7 min	SPM12, DPABI, REST	DC	0.01–0.08	6	*P* <0.01 (FDR corrected)	No	17
Li et al. (17)	3.0 T	>5 min	SPM12, REST	DC	0.01–0.08	6	*P* <0.05 (GRF corrected)	No	17
Liu et al. (123)	3.0 T	8 min	SPM8, DPARSF	ReHo	0.01–0.08	8	*P* <0.01 (AlphaSim corrected)	No	17
Xiong et al. (26)	3.0 T	8 min	SPM12, DPABI	zReHo	0.01–0.1	unknown	*P* <0.01 (AlphaSim corrected)	No	18
Feng et al. (16)	3.0 T	>6 min	SPM8, DPARSF	DC	0.01–0.08	6	*P* <0.05 (FWE corrected)	Yes	19

GMV, gray matter volume; SPM, statistical parametric mapping; DPARSF, data processing assistant for resting-state fMRI; REST, the resting-state fMRI data analysis toolkit; DPABI, data processing and analysis for brain imaging; AFNI, analysis of functional neuroimages; ReHo regional homogeneity; mReHo, mean regional homogeneity; zReHo, ReHo after z-transformation; ICA, independent component analysis; DC, degree centrality; FDR, false discovery rate; FWE, family-wise error; GRF, Gaussian random field.

a Maximum score of 20 for each study.

b Data set of the indicator of degree centrality (DC).

c Data set of the indicator of mean regional homogeneity (mReHo).

**Table 3:** Demographic, clinical, and cognitive characteristics of study patients and control subjects included in the meta-analysis (group 2).

**Study**	**Sex (male/female)**		**Mean Age (SD)**		**Education years (SD)**		**T2DM duration**		**HbA1c (%) (SD)**		**MMSE (SD)**		**MoCA (SD)**	
							**(SD) in years**							
	**T2DM**	**HC**	**T2DM**	**HC**	**T2DM**	**HC**	**T2DM**	**HC**	**T2DM**	**HC**	**T2DM**	**HC**	**T2DM**	**HC**
Xia et al. (23)	15/13	13/16	58.7 (8.1)	57.7 (7.2)	9.9 (3.7)	11.0 (2.0)	9.8 (5.5)	–	7.9 (1.7)	5.6 (0.4)	–	–	23.2 (3.1)	24.1 (2.6)
Wang et al. (20)	17/9	17/9	54.7 (10.4)	54.9 (9.8)	11.2 (3.8)	10.7 (3.2)	8.7 (6.3)	–	8.3 (1.4)	–	27.8 (2.5)	28.3 (1.3)	23.7 (3.0)	26.0 (2.8)
Cui et al. (10)	14/15	11/16	58.3 (7.3)	57.8 (5.9)	10.4 (4.0)	10.2 (2.5)	9.3 (3.8)	–	7.9 (1.7)	5.6 (0.4)	28.3 (1.4)	29.0 (1.1)	23.6 (2.9)	27.3 (1.1)
Xia et al. (124)	17/21	21/19	56.0 (6.1)	57.1 (7.6)	9.6 (3.0)	10.3 (1.9)	7.1 (3.5)	–	7.2 (1.1)	5.6 (0.3)	29.0 (0.9)	29.1 (1.0)	–	–
Liu^a^ et al. (60)	14/8	10/13	58.5 (9.4)	58.6 (8.7)	11.2 (2.7)	10.2 (2.9)	–	–	8.1 (1.5)	5.6 (0.4)	27.9 (1.3)	27.8 (1.4)	–	–
Liu^b^ et al. (24)	12/9	10/15	61.7 (5.3)	58.0 (7.9)	9.8 (2.7)	10.1 (2.8)	7.9 (5.5)	–	9.6 (2.6)	–	28.5 (1.5)	27.7 (1.4)	21.8 (1.4)	26.6 (0.8)
Cui et al. (12)	21/19	13/28	60.5 (6.9)	57.9 (6.5)	10.0 (3.4)	10.3 (2.3)	8.9 (5.0)	–	7.7 (1.6)	5.6 (0.3)	28.3 (1.0)	28.6 (1.2)	–	–
Dai et al. (13)	19/22	16/16	65.5 (8.3)	67.3 (10.1)	15.4 (3.8)	16.1 (3.0)	9.9 (7.9)	–	7.3 (1.3)	5.7 (0.3)	28.6 (1.5)	28.9 (1.6)	–	–
Yu et al. (14)	28/5	22/11	53.5 (8.4)	51.0 (5.3)	12.8 (2.4)	12.9 (3.5)	7.1 (5.2)	–	8.1 (1.7)	5.6 (0.3)	28.9 (0.9)	28.5 (1.1)	26.5 (2.1)	26.8 (2.0)
Liu et al. (27)	24/13	17/20	57.6 (7.1)	57.9 (5.7)	11.6 (3.9)	10.9 (2.3)	8.7 (5.5)	–	7.6 (1.5)	5.7 (0.4)	28.0 (1.5)	28.5 (1.2)	22.5 (2.7)	24.2 (2.7)

Data are presented as mean (SD), or ratios. T2DM, type 2 diabetes mellitus; HC, healthy control; SD, standard deviation; HbA1c, glycosylated hemoglobin A1c; MMSE, mini-mental state examination; MoCA, montreal cognitive assessment.

a The study topic is sub-band analysis of amplitude of low-frequency fluctuations in type 2 diabetes mellitus patients: a resting-state functional MRI study.

b The study topic is spontaneous brain activity alterations in T2DM patients with mild cognitive impairment: a resting-state fMRI study.

**Table 4:** Imaging characteristics of the studies included in the meta-analysis (group 2).

**Study**	**Scanner**	**Scan**	**Software**	**Indicator**	**Frequency**	**Smoothing**	**Threshold**	**GMV**	**Quality**
		**duration**			**range (Hz)**	**kernel (mm)**	**(method)**	**correction**	**scores^a^**
Xia et al. (23)	3.0 T	> 6 min	SPM8, DPARSF, REST	ALFF	0.01–0.08	4	*P* <0.05 (AlphaSim corrected)	No	19
Wang et al. (20)	3.0 T	7 min	SPM8, DPARSF, REST	ALFF	0.01–0.08	4	*P* <0.01 (AlphaSim corrected)	Yes	18
Cui et al. (10)	3.0 T	> 6 min	SPM8, DPARSF, REST	ALFF	0.01–0.08	4	*P* <0.01 (AlphaSim corrected)	Yes	17
Xia et al. (124)	3.0 T	45 min^d^	SPM8	ASL	–	6	*P* <0.01 (FWE corrected)	Yes	19
Liu^b^ et al. (60)	3.0 T	8 min	SPM8, DPARSF, REST	fALFF	0.01–0.027	4	*P* <0.05 (AlphaSim corrected)	No	17
Liu^b^ et al. (60)	3.0 T	8 min	SPM8, DPARSF, REST	fALFF	0.027–0.073	4	*P* <0.05 (AlphaSim corrected)	No	17
Liu^c^ et al. (24)	3.0 T	8 min	SPM8, DPARSF, REST	fALFF	0.01–0.08	4	*P* <0.05 (AlphaSim corrected)	No	17
Cui et al. (12)	3.0 T	8 min	SPM8, AFNI	ASL	–	6	*P* <0.05 (AlphaSim corrected)	No	18
Dai et al. (13)	3.0 T	unknown	SPM8	ASL	–	unknown	*P* <0.5 (FWE corrected)	Yes	17
Yu et al. (14)	3.0 T	> 6 min	SPM8, DPABI, REST	ASL	–	8	*P* <0.05 (GRF corrected)	Yes	20
Liu et al. (27)	3.0 T	unknown	SPM8, DPABI	ALFF	0.01–0.1	4	*P* <0.05 (GRF corrected)	No	18

GMV, gray matter volume; SPM, statistical parametric mapping; DPARSF, data processing assistant for resting-state fMRI; REST, resting-state fMRI data analysis toolkit; DPABI, data processing and analysis for brain imaging; FDG, fluoro-deoxy-glucose; PMOD, PMOD Technologies, Ltd., Zurich, Switzerland; AFNI, analysis of functional neuroimages; ALFF, amplitude of low-frequency fluctuation; fALFF, fractional ALFF; PET, positron emission tomography; ASL arterial spin labeling; FWE, family-wise error; GRF, Gaussian random field.

a Maximum score of 20 for each study.

b Study topic is sub-band analysis of amplitude of low-frequency fluctuations in type 2 diabetes mellitus patients: a resting-state functional MRI study.

c Study topic is spontaneous brain activity alterations in T2DM patients with mild cognitive impairment: a resting-state fMRI study.

d The entire scan lasted 45 min and included perfusion, anatomic, and other scanning oxygen level-dependent and diffusion tensor imaging.

### Abnormal function of the brain area obtained by PSI-SDM

**Figure 2:**
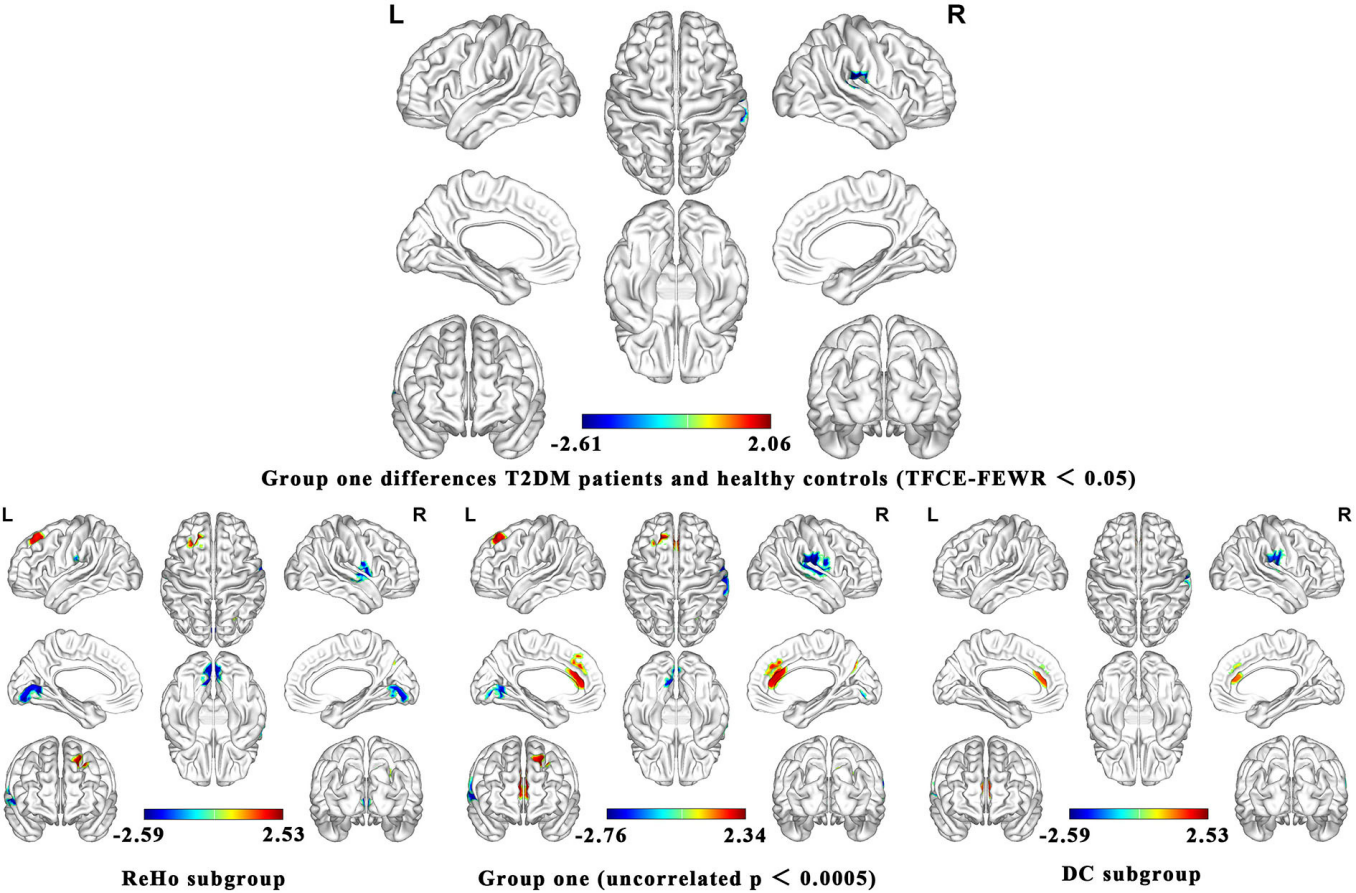
Meta-analysis results of the all 17 data sets comparing group 1 differences between T2DM patients and healthy controls.

As shown in Figure 2, the brain functional activity of the right Roland tegmentum, right superior marginal gyrus, and right superior temporal gyrus in T2DM patients compared with HCs decreased in group 1 (p_TFCE−FEW_ = 0.018, effect size = −0.31, CI = [−0.40, −0.23], cluster = 149 voxels), while there was no significant difference after correction in group 2. The results of PSI-SDM are summarized in Table 5.

**Table 5:** Clusters of group 1 differences in patients with T2DM compared to healthy controls.

	**Brain region**	**MNI**	***P*-value**	**Number of**	**SDM-Z**	**Egger's test**
	**and cluster breakdown**	**coordinate**		**Voxels**	**value**	***p-*value)**
Decreased in Group one	Right Rolandic operculum (BA 48)	56, −30, 22	0.018	164	−4.277	0.870
	Right supramarginal gyrus	64, −22, 18				
	Right superior temporal gyrus	62, −30, 18				

### Uncorrected results and subgroup analysis are obtained by AES-SDM

In group 1, the indicators of brain function combining ReHo, ICA, and DC increased in the left superior frontal gyrus and right precuneus, while decreased in the right postcentral gyrus, left lingual gyrus, and right supramarginal gyrus in T2DM patients compared with HCs. In group 2, the indicators including ALFF and CBF increased in the right and left anterior cingulate/paracingulate gyri, while decreased in the right linguistic gyrus and right middle occipital gyrus in T2DM patients compared with HCs.

We analyzed five subgroups, namely, ReHo, ICA, DC, ALFF, and CBF. The subgroup analysis showed that it was feasible to divide the functional imaging methods into two groups, which reflected the relationship of nerve activity and the intensity of nerve activity. The results of ReHo and DC were consistent to some extent (Figure 2). There was no significant difference in ICA between T2DM patients and healthy people. Since there are only two studies in ICA, the results are not shown in Figure 2. The details of the analytical results were found in Supplementary Tables S3, S4, Supplementary Figures S2, S3.

### Analyses of heterogeneity and publication bias

**Figure 3:**
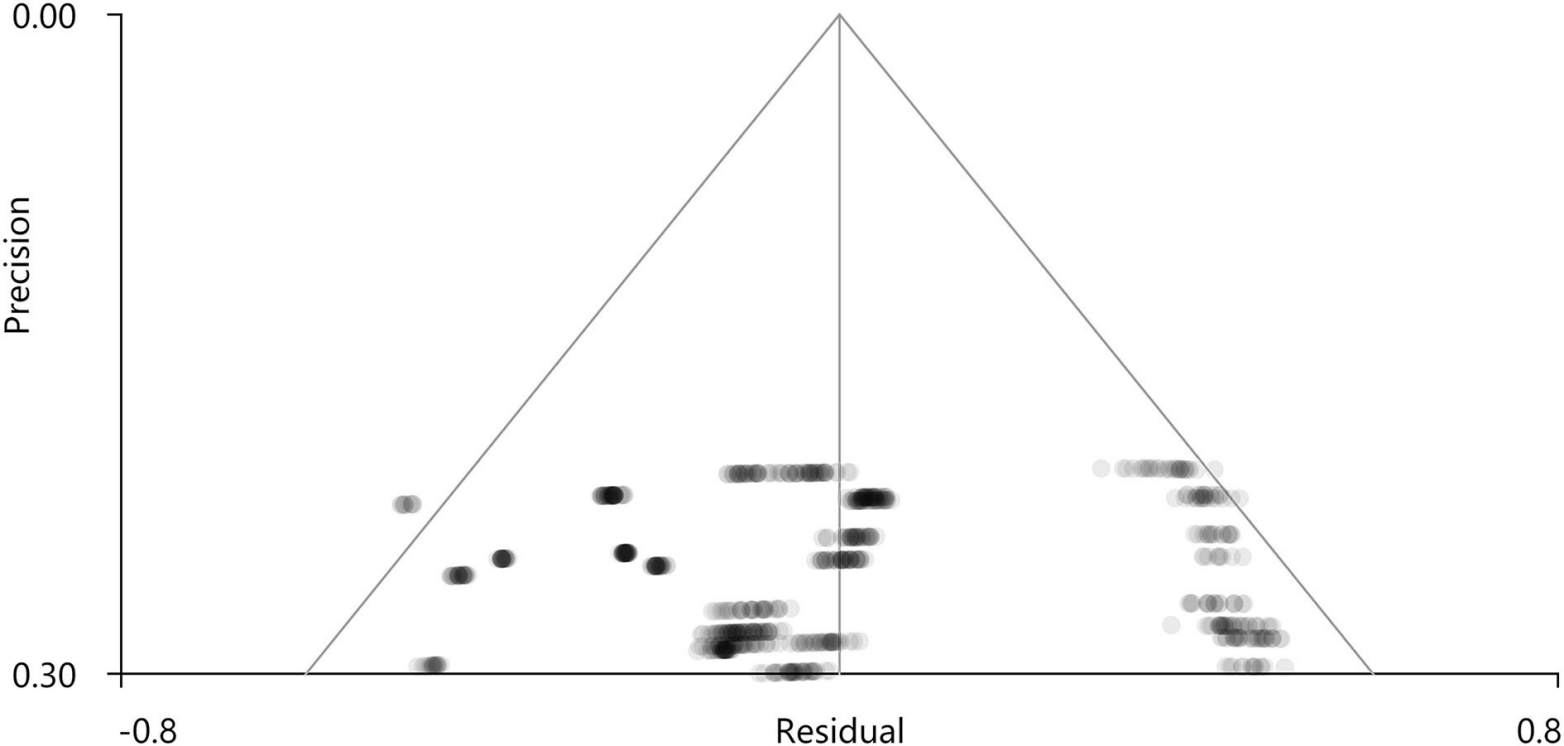
Funnel plot of effect size of the right supramarginal gyrus.

**Figure 4:**
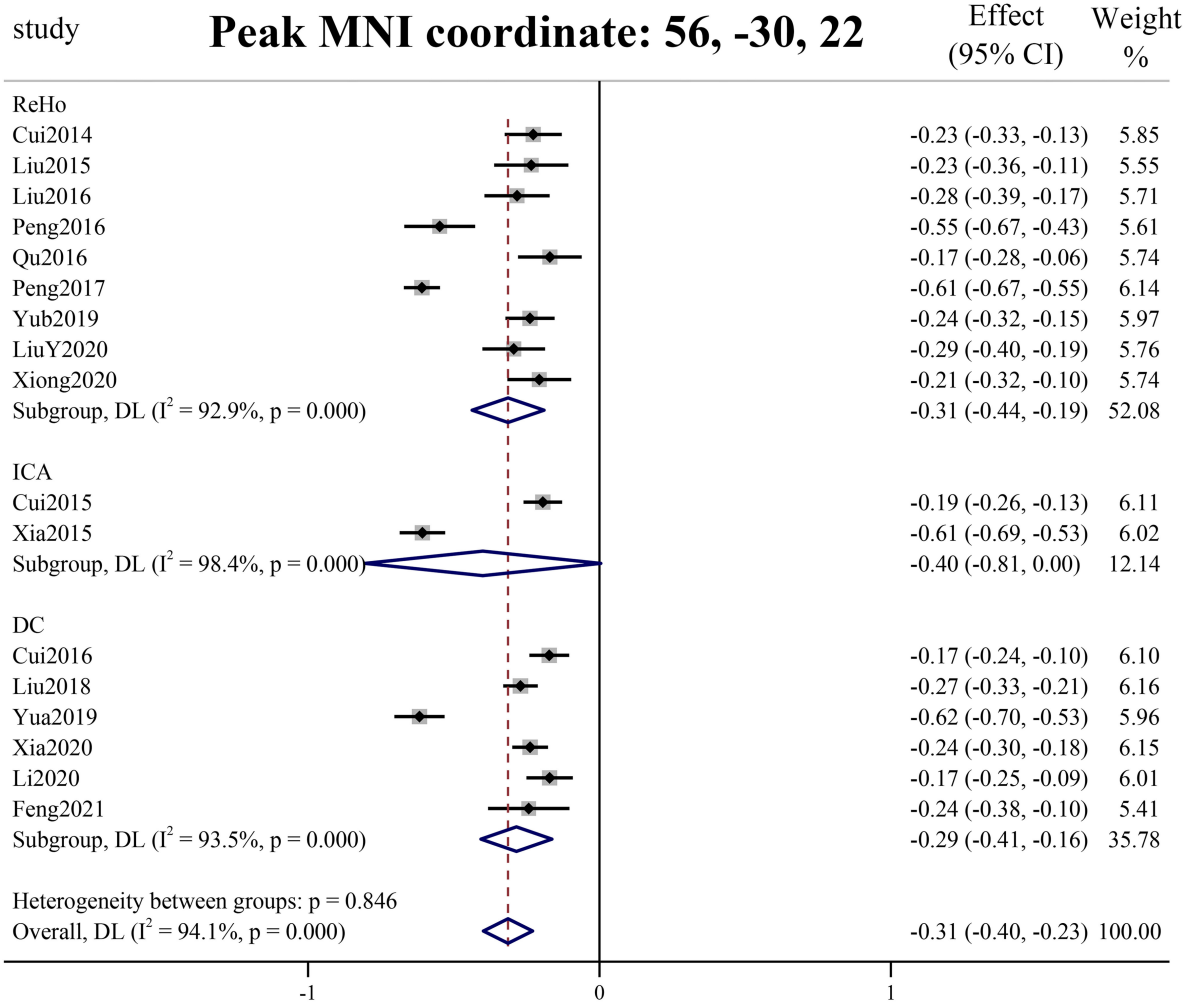
Forest plot of peak MNI coordinate.

PSI-SDM software used Q and H tests to assess heterogeneity in the right Rolandic operculum (peak MNI coordinate: x = 56, Y = −30, Z = 22, *p* = 0.018, Z = −4.277, 164 voxels). But the results are inconsistent. Thus, we used Stata software to evaluate its heterogeneity. The results showed that there was significant heterogeneity (SDM = −0.31, 95% CI = [−0.40, −0.23], I2 = 94.1%), and the effect size obtained by the random-effects model was consistent with PSI-SDM (SDM = −0.31, variance = 0.005362, Z = −4.277). The forest plot is given in Supplementary Figure S4. Our study had no publication bias (see Supplementary Table S5, Supplementary Figure S5), as revealed by the funnel plot showing approximate symmetry (Figure 3). In addition, it was observed that Egger's test (*P* = 0.870) detected no publication bias in our meta-analysis (Table 5). Finally, we used Stata software to evaluate the heterogeneity of group 1. The heterogeneity between ReHo, ICA, and DC subgroups is not mainly determined by different brain function processing strategies (Figure 4).

### Jackknife sensitivity analysis

We performed sensitivity analysis based on AES-SDM. After preprocessing, the whole-brain voxel sensitivity analysis was performed by repeating the same analysis iteratively, eliminating one data set at a time to test the reproducibility of the results. The results are shown in Table 6.

**Table 6:** Sensitivity analysis of the group 1.

**Study**	**R PCG**	**R ROL**	**L LG**	**L SFG**	**R PCu**
Cui et al. (10)	√	√	×	√	×
Cui et al. (15)	√	√	√	√	√
Xia et al. (21)	√	√	√	√	√
Liu et al. (24)	√	√	√	√	√
Cui et al. (11)	√	√	√	√	√
Liu et al. (18)	√	√	√	√	√
Peng et al. (120)	√	√	×	√	×
Qu et al. (121)	√	√	×	×	×
Peng et al. (122)	√	√	×	×	×
Liu et al. (19)	√	√	√	√	√
Yu^a^ et al. (14)	√	√	√	√	√
Yu^b^ et al. (14)	√	√	√	√	√
Xia et al. (25)	√	√	√	√	√
Li et al. (17)	√	√	√	√	√
Liu et al. (123)	√	√	√	√	√
Xiong et al. (26)	√	√	√	√	√
Feng et al. (16)	√	√	√	√	√
Total	17 out of 17	17 out of 17	13 out of 17	15 out of 17	13 out of 17

R, right; L, left; PCG, postcentral gyrus; ROL, Rolandic operculum; LG, lingual gyrus; SFG, superior frontal gyrus; PCu, precuneus. Our sensitivity analysis was based on AES-SDM.

a Data set of the indicator of degree centrality (DC).

b Data set of the indicator of mean regional homogeneity (mReHo).

### Intra-class correlation analysis

**Figure 5:**
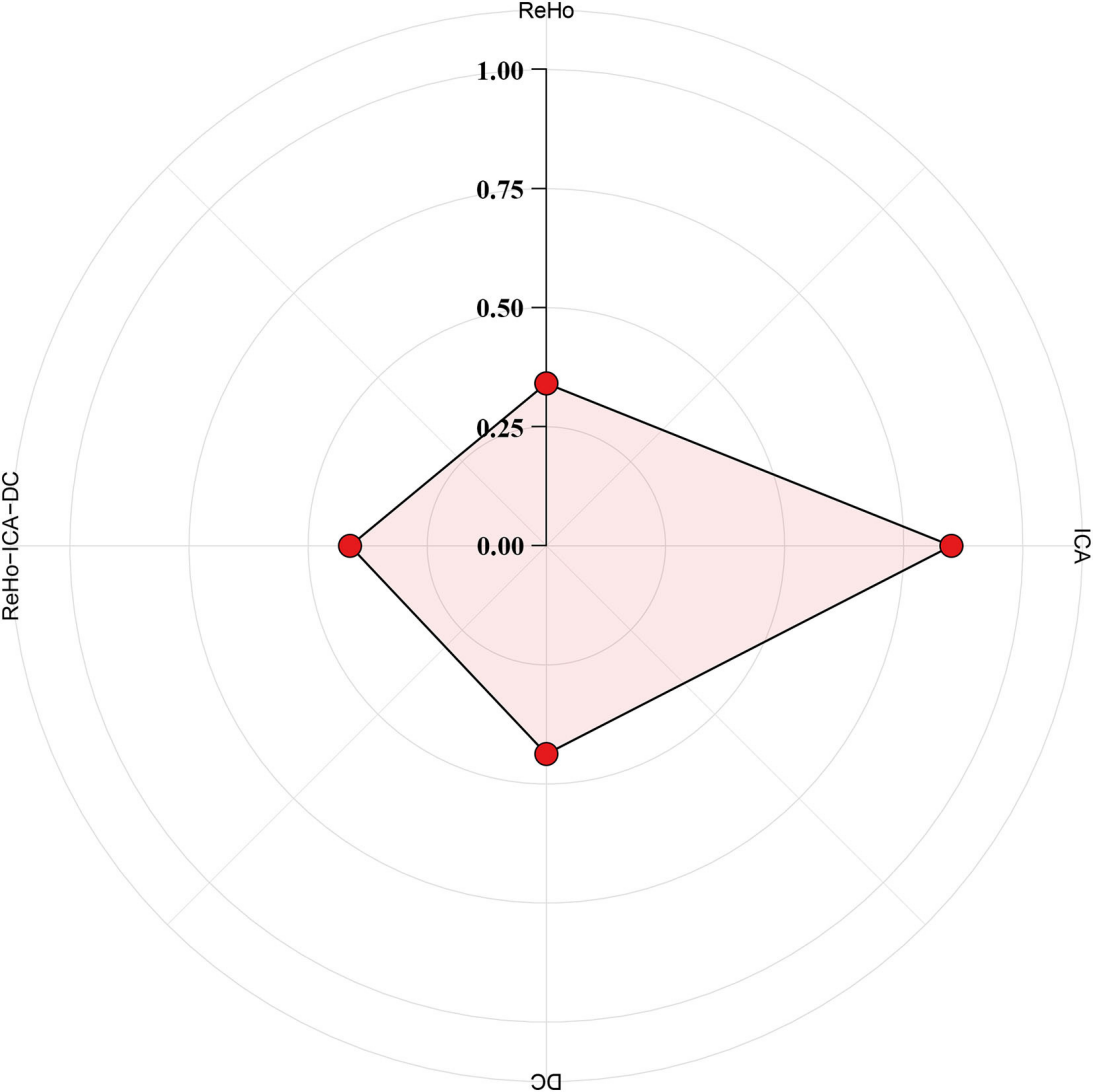
Indicator of reliable brain function. The test–retest reliability was measured with intra-class correlation (ICC) for the following brain functional indicator: regional homogeneity (ReHo), independent component analysis (ICA), degree centrality (DC), and ReHo, ICA, and DC combined. A metric with moderate to almost perfect test–retest reliability (ICC ≥ 0.4) is commonly expected in practice.

The ICC analysis were performed by SPSS. In group 1, the ICC scores of ReHo, ICA, and DC were 0.341, 0.850, and 0.437, respectively, and 0.412 when combining indicators of ReHo, ICA, and DC. The results of ICC are summarized in Figure 5.

### Meta-regression

The meta-regression analysis showed that general demographic data had no significant effect on the main results. The lower MoCA scores in patients with T2DM were associated with greater decreases in the brain function indicator in the bilateral anterior cingulate/paracingulate gyrus (peak MNI coordinate: x = 2, y = 32, z = 24, *p* = 0.0001, Z = −1.881, 149 voxels).

## Discussion

The quantitative meta-analysis of whole-brain different indexes of brain functions demonstrated differences between T2DM patients and healthy controls *via* PSI-SDM. The results of meta-analysis showed that the brain function indexes of group 1 had a decreased function in the right Rolandic operculum, right supramarginal gyrus, and right superior temporal gyrus. The jackknife sensitivity analysis demonstrated that these peak coordinates were highly reproducible. However, the subgroup analysis did not have significant peak coordinates, possibly due to inadequate data sets and over-conservative corrections.

Neurobiology and neuroimaging studies indicated that decreased right Rolandic operculum (ROL.R) function is associated with emotional processing such as depression, apathy, and anxiety (9, 33, 83, 125, 126). A recent neuroimaging systematic review highlighted the need to assess cognitive and emotional impairments in T2DM patients, which may increase the risk of mental health (127). Previous meta-analysis by AES-SDM software also concluded that T2DM patients had decreased brain function in the ROL.R region (33), which is highly consistent with our meta-analysis. Herein, we speculate that T2DM patients may have increased risk of emotional disorders due to damage in the ROL.R area. On the other hand, another meta-analysis did not find abnormal ROL.R area, which may be due to the use of all brain imaging strategies (28). In addition to emotional processing, ROL.R plays a role in the sensory system of visceral sensation together with the cingulate–operculum network (125, 128, 129). Visceral sensation disorders may be related to psychological states, such as depression, anxiety, and stress (125). Negative emotions trigger cortisol release to induce local immune activity, thereby changing intestinal permeability and lowering probiotic levels (125, 130). Moreover, the relationship between the brain and gastrointestinal organs could be explained *via* the brain–gut axis theory (129). Previous studies had also shown that T2DM might cause cognitive and emotional impairment through the brain–gut axis (131, 132). Thus, future studies on T2DM-induced MCI and emotional disorders can focus on the brain–gut axis or ROL.R-related network connectivity in future.

Beyond the ROL.R region in the cingulate–operculum network, our meta-analysis also observed decreased brain activity in the right supramarginal gyrus (SMG.R). The SMG.R is involved in second-language acquisition and is essential in learning and remembering (133). Social cognition studies have shown that the SMG.R also has certain attention, memory, language, and social behavior abilities (133). Recent studies have shown that the SMG.R may be related to visuospatial disorders and is essential in spatial orientation and semantic arrangement (134). Visual spatial orientation disorder is also one of the manifestations of MCI (135). The meta-analysis results of Li et al. (33) showed that the gray matter volume of T2DM and healthy control groups decreased in the SMG. R. The structural changes are consistent with the results of our meta-analysis. Some studies have also reported that the ReHo value is significantly reduced in the SMG.R between MCI patients and HCs (134). Therefore, T2DM patients may have MCI, which increases the risk of dementia. In addition, we observed decreased spontaneous brain activity in the right superior temporal gyrus, which was an important brain alternation for patients with depression (136). Consistent with our research, some meta-analyses showed that the gray matter volume (GMV) and neural activity of the superior temporal gyrus decreased significantly in T2DM patients, which may be related to emotional disorders (33, 137, 138). In future, the effects of depression on the brain structure and function of patients with diabetes should be consider independently.

Subgroup analysis showed that both ReHo and ICA/DC subgroups found decreased brain function in the postcentral gyrus and ROL.R in T2DM patients. It should be noted that the ROL.R was an important brain region after correction. This shows that ReHo and ICA/DC subgroups are consistent with the conclusions of the main analysis. Sensitivity analysis also proved the stability and repeatability of the ROL.R region. In addition, our meta-regression analysis showed that the severity of cognitive impairment was related to the activity of the bilateral anterior cingulate/paracingulate gyrus. The bilateral anterior cingulate/paracingulate gyrus was involved in the default mode network and was highly related to cognitive and emotional disorders (139, 140). Previous studies identified that T2DM is an important risk factor for MCI and can accelerate the progression of patients to dementia (141). In future, we need to further explore the differences in brain functions between T2DM with MCI and without MCI. However, this study had a high heterogeneity. Therefore, a subgroup analysis on the effect size was conducted using a general meta-analysis method to assess the causes of heterogeneity. The results showed that the difference between ReHo and ICA/DC was not the primary source of heterogeneity. Previous studies have demonstrated that comprehensive background such as the age, course of disease, education level, emotional disorder, and biomarkers like HbA1c were related to cognitive impairment (142, 143). In addition, the brain regions of cortical atrophy in patients with T2DM were also found in early Alzheimer's disease (144). The size of the GMV affects the brain function and cognitive level in T2DM (144). Therefore, we speculated that the education level, HbA1c, T2DM duration, and whole-brain mean GMV could have caused the heterogeneity. We used meta-regression analysis to show that the education level, HbA1c, and T2DM duration had no significant impact on the results. However, we did not have adequate data to correct the GMV. Therefore, we speculated that GMV was the main cause of heterogeneity in our meta-analysis.

The ICC analysis showed that the combined ReHo, ICA, and DC was reliable, and the individual differences within group are acceptable. A previous study found that ReHo, ICA, and VMHC are most reliable across examined voxel-wise metrics (145, 146). An ICC score ≥ 0.4 was considered to have moderate to almost perfect test–retest reliability (145). In our study, the reliability of ReHo is low, which may be caused by different degrees of preprocessing, scan duration, imaging resolution, and computational space (147). Although we have analyzed the reliability and validity of individual difference measurement, our evaluation is still unsatisfactory. It is believed that neuroimaging researchers may be collecting larger amounts of suboptimal data, rather than smaller amounts of higher quality data, and this does not bode well for the field, particularly when it comes to information and predictions at the individual level (135). Therefore, this is both an opportunity and a challenge for our research work in future. We will devote ourselves to that, this misstep can be avoided by critical assessments of reliability upfront.

Furthermore, our meta-analysis results of T2DM patients were inconsistent with those of the recent meta-analysis of rs-fMRI studies on T2DM using activation likelihood estimation (ALE) (28). Xia et al. found that T2DM patients have decreased functions in the left and the right lingual gyrus, left posterior central gyrus, right inferior temporal gyrus, right cerebellar culmen, right insular lobe, and right posterior cingulate cortex and enhanced functions in the right precuneus and left superior frontal gyrus (28). This inconsistency may be attributed to the following limitations of the ALE meta-analysis (29, 42, 44). First, ALE meta-analysis combines several methods of analyzing brain function based on different principles, making the results difficult to interpret (29, 31). Due to the limited number of studies on all analytic approaches of brain function, in the current study, we divided different brain imaging strategies into two categories, which is helpful to better understand the aberrant spontaneous brain activity in T2DM. Second, ALE does not develop sensitivity, heterogeneity, publication bias, and meta-regression analysis (31, 39). The PSI-SDM and AES-SDM used herein adopted and combined various positive features from other existing meta-analysis methods, such as ALE or multilevel kernel density analysis, and introduced several improvements and new features (42, 44). Another meta-analysis of abnormal brain areas of ALFF in patients with MCI showed that integrating different analysis approaches and modalities is not optimal (31). However, the meta-analysis of single indicator of neuroimaging also has the limitation of the number of studies. Thus, our study indicates that we can divide the different functional imaging methods into reflecting intensity and reflecting relationship groups when studies are scarce. But we must carefully interpret the results.

The results obtained by Li et al. using AES-SDM are consistent with our results to some extent (29). However, they are based on AES-SDM and have some drawbacks because the AES-SDM analysis results were not corrected (42, 44). They found out the bilateral superior temporal gyrus/Rolandic operculum, left middle and inferior temporal gyrus, left supramarginal gyrus, the cerebellum, insula, and visual cortex dysfunction in patients with T2DM (33). Our meta-analysis did not reveal functional abnormalities of T2DM in the left middle and infernal temporal gyrus, left supratrigonal gyrus, the cerebellum, insula, and visual cortex. We believe that the recent finding based on AES-SDM that T2DM has a wide range of default-mode network anomalies may not be completely accurate (33, 42, 44). There is no evidence that T2DM patients have a wide range of default mode network anomalies. We need to study the spontaneous abnormal brain function of patients with T2DM more deeply.

Finally, this meta-analysis also had some limitations. First, like other methods in a coordinate-based meta-analysis, SDM was based on the coordinates of the published study instead of the raw data, limiting its accuracy (40, 42). Second, the meta-analysis mainly included Asian samples, limiting its universality. Third, the current meta-analysis could not determine a causal relationship between T2DM and abnormal activity in brain regions since all studies included were cross-sectional (40). Fourth, although we speculated that heterogeneity in the meta-analysis might be due to the GMV, there were insufficient data to calibrate the results with GMV as a covariate.

## Conclusion

Our comprehensive meta-analysis showed that T2DM had a range of spontaneous abnormal brain activities, mainly involved in brain regions associated with learning, memory, and emotion, which is helpful to understand the neuropathophysiological mechanism of T2DM. Although the results of single index meta-analysis may be more explanatory, the repeatability of the results of meta-analysis will be very low when there are <10 studies on the same indicator. Therefore, our team applied a strategy which divided the different functional imaging processing methods into two groups, namely, reflecting the intensity of neural activity in the brain and the relationship of neural activity in the brain. We found that the abnormal regions of different indexes have a certain consistency and high stability and repeatability. The main reason for the first group of heterogeneity was not caused by differences in indexes of brain function. Although ReHo, ICA, and DC reflect the relationship of neural activity in the brain, their heterogeneity cannot be ignored. Therefore, we suggest that the results of a meta-analysis of a single index may be more explicable when ample studies are included. Conversely, when studies are scarce, we can divide the different functional imaging processing methods into reflecting intensity and reflecting relationship groups, but we must interpret the results carefully. We believe that this strategy is more reliable than a meta-analysis combining all brain function processing methods, but more in-depth research is needed.

## Data availability statement

The original contributions presented in the study are included in the article/Supplementary material, further inquiries can be directed to the corresponding author/s.

## Author contributions

G-BC, L-FY, and WW contributed to the conception of the study. Z-YL, TM, Y-MZ, YY, and BH performed the experiment and collected data. Z-YL, TM, Y-MZ, YH, HX, and M-HN contributed significantly to analysis and manuscript preparation. Z-YL, TM, Y-MZ, and L-FY performed the data analyses and wrote the manuscript. Z-HC, Y-XH, and W-HL helped perform the analysis with constructive discussions. All authors contributed to the article and approved the submitted version.

## Funding

This work was supported by the National Natural Science Foundation of China [Grant number 81771815].

## Conflict of interest

The authors declare that the research was conducted in the absence of any commercial or financial relationships that could be construed as a potential conflict of interest.

## Publisher's note

All claims expressed in this article are solely those of the authors and do not necessarily represent those of their affiliated organizations, or those of the publisher, the editors and the reviewers. Any product that may be evaluated in this article, or claim that may be made by its manufacturer, is not guaranteed or endorsed by the publisher.
