# Supplementary material for: Changes of brain function in patients with type 2 diabetes mellitus measured by different analysis methods: A new coordinate-based meta-analysis of neuroimaging
Source: Front Neurol. 2022 Aug 24;13:923310. doi: 10.3389/fneur.2022.923310 (PMC9449648; doi:10.3389/fneur.2022.923310)
Supplement: Supplementary file 1 [file Data_Sheet_1.pdf]

Supplementary Materials

Changes of brain function in patients with type 2 diabetes mellitus measured by different analysis method: A new coordinate based meta-analysis of neuroimaging

Contents

Table S1. Checklist of the PRISMA extension..2

Text S1. The detailed search strategy for the database 8

Table S2. Checklist for objective assessment of methodological quality of studies for neuroimaging meta-analysis 14

Figure S1. objective assessment of quality of 23 studies 16

Table S3 Meta-analytic results of the group one and differences between subgroups 17

FigureS2. Similarities and differences between the group one analysis and the subgroups analysis. 17

Table S4 Meta-analytic results of the group two and results of subgroup analysis 17

FigureS3 Similarities and differences between the group two analysis and the subgroup analysis 18

FigureS4. Forest plot of meta-analysis for effect size of right rolandic operculum 18

TableS5. Egger's test 19

FigureS5. Egger's test 19

TableS1. Checklist of the PRISMA extension

Section and Topic	Item #	Checklist item	Location where item is reported
**TITLE**			
Title	1	Identify the report as a systematic review.	**1**
**ABSTRACT**			
Abstract	2	Provide a structured summary including, as applicable: background; objectives; data sources; study eligibility criteria, participants, and interventions; study appraisal and synthesis methods; results; limitations; conclusions and implications of key findings; systematic review registration number.	**1-2**
**INTRODUCTION**			
Rationale	3	Describe the rationale for the review in the context of existing knowledge.	**3-5**
Objectives	4	Provide an explicit statement of the objective(s) or question(s) the review addresses.	**6**
**METHODS**			
Eligibility criteria	5	Specify the inclusion and exclusion criteria for the review and how studies were grouped for the syntheses.	**7-8**
Information sources	6	Specify all databases, registers, websites, organisations, reference lists and	**7-8**

Section and Topic	Item #	Checklist item	Location where item is reported
		other sources searched or consulted to identify studies. Specify the date when each source was last searched or consulted.	
Search strategy	7	Present the full search strategies for all databases, registers and websites, including any filters and limits used.	**Supplementary Materials**
Selection process	8	Specify the methods used to decide whether a study met the inclusion criteria of the review, including how many reviewers screened each record and each report retrieved, whether they worked independently, and if applicable, details of automation tools used in the process.	**8**
Data collection process	9	Specify the methods used to collect data from reports, including how many reviewers collected data from each report, whether they worked independently, any processes for obtaining or confirming data from study investigators, and if applicable, details of automation tools used in the process.	**8**
Data items	10a	List and define all outcomes for which data were sought. Specify whether all results that were compatible with each outcome domain in each study were sought (e.g. for all measures, time points, analyses), and if not, the methods used to decide which results to collect.	**9-11**
	10b	List and define all other variables for which data were sought (e.g. participant and intervention characteristics, funding sources). Describe any assumptions made about any missing or unclear information.	**9-11**

Section and Topic	Item #	Checklist item	Location where item is reported
Study risk of bias assessment	11	Specify the methods used to assess risk of bias in the included studies, including details of the tool(s) used, how many reviewers assessed each study and whether they worked independently, and if applicable, details of automation tools used in the process.	**12-13**
Effect measures	12	Specify for each outcome the effect measure(s) (e.g. risk ratio, mean difference) used in the synthesis or presentation of results.	**11**
Synthesis methods	13a	Describe the processes used to decide which studies were eligible for each synthesis (e.g. tabulating the study intervention characteristics and comparing against the planned groups for each synthesis (item #5)).	**Table1-Table4**
	13b	Describe any methods required to prepare the data for presentation or synthesis, such as handling of missing summary statistics, or data conversions.	**10-11**
	13c	Describe any methods used to tabulate or visually display results of individual studies and syntheses.	**10-12**
	13d	Describe any methods used to synthesize results and provide a rationale for the choice(s). If meta-analysis was performed, describe the model(s), method(s) to identify the presence and extent of statistical heterogeneity, and software package(s) used.	**10-12**
	13e	Describe any methods used to explore possible causes of heterogeneity among study results (e.g. subgroup analysis, meta-regression).	**10-12**

Section and Topic	Item #	Checklist item	Location where item is reported
	13f	Describe any sensitivity analyses conducted to assess robustness of the synthesized results.	**12**
Reporting bias assessment	14	Describe any methods used to assess risk of bias due to missing results in a synthesis (arising from reporting biases).	**11-12**
Certainty assessment	15	Describe any methods used to assess certainty (or confidence) in the body of evidence for an outcome.	**NA**
**RESULTS**			
Study selection	16a	Describe the results of the search and selection process, from the number of records identified in the search to the number of studies included in the review, ideally using a flow diagram.	**12, Figure 1**
	16b	Cite studies that might appear to meet the inclusion criteria, but which were excluded, and explain why they were excluded.	**12-13**
Study characteristics	17	Cite each included study and present its characteristics.	**12-14**
Risk of bias in studies	18	Present assessments of risk of bias for each included study.	**Supplementary Materials**
Results of individual studies	19	For all outcomes, present, for each study: (a) summary statistics for each group (where appropriate) and (b) an effect estimate and its precision (e.g. confidence/credible interval), ideally using structured tables or plots.	**Table1-Table4 Supplementary Materials**

Section and Topic	Item #	Checklist item	Location where item is reported
Results of syntheses	20a	For each synthesis, briefly summarise the characteristics and risk of bias among contributing studies.	**Supplementary Materials**
	20b	Present results of all statistical syntheses conducted. If meta-analysis was done, present for each the summary estimate and its precision (e.g. confidence/credible interval) and measures of statistical heterogeneity. If comparing groups, describe the direction of the effect.	**13-15**
	20c	Present results of all investigations of possible causes of heterogeneity among study results.	**16-18**
	20d	Present results of all sensitivity analyses conducted to assess the robustness of the synthesized results.	**16-18**
Reporting biases	21	Present assessments of risk of bias due to missing results (arising from reporting biases) for each synthesis assessed.	**16-18**
Certainty of evidence	22	Present assessments of certainty (or confidence) in the body of evidence for each outcome assessed.	**NA**
**DISCUSSION**			
Discussion	23a	Provide a general interpretation of the results in the context of other evidence.	**19**
	23b	Discuss any limitations of the evidence included in the review.	**19-21**
	23c	Discuss any limitations of the review processes used.	**22**

Section and Topic	Item #	Checklist item	Location where item is reported
	23d	Discuss implications of the results for practice, policy, and future research.	**21-23**
**OTHER INFORMATION**			
Registration and protocol	24a	Provide registration information for the review, including register name and registration number, or state that the review was not registered.	**6**
	24b	Indicate where the review protocol can be accessed, or state that a protocol was not prepared.	**6**
	24c	Describe and explain any amendments to information provided at registration or in the protocol.	**NA**
Support	25	Describe sources of financial or non-financial support for the review, and the role of the funders or sponsors in the review.	**23-26**
Competing interests	26	Declare any competing interests of review authors.	**23-26**
Availability of data, code and other materials	27	Report which of the following are publicly available and where they can be found: template data collection forms; data extracted from included studies; data used for all analyses; analytic code; any other materials used in the review.	**NA**

TextS1. The detailed search strategy for the database

PubMed:

(((((((Diabetes Mellitus, Type 2[MeSH Terms]) OR (Diabetes Mellitus, Type 2[Text Word])) OR (Type 2 Diabetes[Text Word])) OR (Diabetes Mellitus, Type II[Text Word])) OR (NIDDM[Text Word])) OR (T2DM[Text Word])) AND (((((((((((functional neuroimaging[MeSH Terms]) OR (functional neuroimaging[Text Word])) OR (Functional Brain Imaging[Text Word])) OR (resting state[Text Word])) OR (default network[Text Word])) OR (functional magnetic resonance imaging[Text Word])) OR (FMRI[Text Word])) OR (ReHo[Text Word])) OR (ALFF[Text Word])) OR ((((((Cerebrovascular Circulation[MeSH Terms]) OR (Cerebrovascular Circulation[Text Word])) OR (Cerebral Blood Flow[Text Word])) OR (CBF[Text Word])) OR (arterial spin labeling[Text Word])) OR (ASL[Text Word]))) OR (((Positron Emission Tomography[MeSH Terms]) OR (Positron Emission Tomography[Text Word])) OR (PET[Text Word])))) AND (((((((Cognitive Dysfunction[MeSH Terms]) OR (Cognitive Dysfunction[Text Word])) OR (Cognitive Impairment[Text Word])) OR (Mild Cognitive Impairment[Text Word])) OR (Cognitive Disorder[Text Word])) OR (MCI[Text Word])) OR (Cognitive Decline[Text Word])) Filters: from 2007/1/1 - 2021/11/15

A total of 163 records

Web Of Science

#1 TOPIC: (Diabetes Mellitus, Type 2) *OR* **TOPIC:** (Type 2 Diabetes) *OR* **TOPIC:** (Diabetes Mellitus, Type II) *OR* **TOPIC:** (T2DM) *OR* **TOPIC:** (NIDDM)

Databases= WOS, CSCD, KJD, MEDLINE, RSCI, SCIELO Timespan=2007-01-01 to 2021-11-15

Search language=Auto

#2 TOPIC: (Cognitive Dysfunction) *OR* **TOPIC:** (Cognitive Impairment) *OR* **TOPIC:** (Cognitive Decline) *OR* **TOPIC:** (Mild Cognitive Impairment) *OR* **TOPIC:** (Cognitive Disorder) *OR* **TOPIC:** (MCI)

Databases= WOS, CSCD, KJD, MEDLINE, RSCI, SCIELO Timespan=2007-01-01 to 2021-11-15

Search language=Auto

#3 TOPIC: (functional neuroimaging) *OR* **TOPIC:** (Functional Brain Imaging) *OR* **TOPIC:** (functional magnetic resonance imaging) *OR* **TOPIC:** (FMRI) *OR* **TOPIC:** (resting state) *OR* **TOPIC:** (default network) *OR* **TOPIC:** (ALFF) *OR* **TOPIC:** (ReHo) *OR* **TOPIC:** (Cerebrovascular Circulation) *OR* **TOPIC:** (cerebral blood flow) *OR* **TOPIC:** (CBF) *OR* **TOPIC:** (ASL) *OR* **TOPIC:** (arterial spin labeling) *OR* **TOPIC:** (Positron Emission Tomography) *OR* **TOPIC:** (PET)

Databases= WOS, CSCD, KJD, MEDLINE, RSCI, SCIELO Timespan=2007-01-01 to 2021-11-15

Search language=Auto

#3 AND #2 AND #1

Databases= WOS, CSCD, KJD, MEDLINE, RSCI, SCIELO Timespan=2007-01-01 to 2021-11-15

Search language=Auto

A total of 480 records

Cochrane Library

ID Search Hits

#1 MeSH descriptor: [Diabetes Mellitus, Type 2] this term only 19039

#2 (Diabetes Mellitus, Type 2):ti,ab,kw OR (Type 2 Diabetes):ti,ab,kw OR (Diabetes Mellitus, Type II):ti,ab,kw OR (T2DM):ti,ab,kw OR (NIDDM):ti,ab,kw 51683

#3 #1or#2 51684

#4 MeSH descriptor: [Functional Neuroimaging] this term only 1877

#5 (functional neuroimaging):ti,ab,kw OR (Functional Brain Imaging):ti,ab,kw OR (functional magnetic resonance imaging):ti,ab,kw OR (FMRI):ti,ab,kw 10511

#6 (resting state):ti,ab,kw OR (default network):ti,ab,kw OR (ALFF):ti,ab,kw OR

(ReHo):ti,ab,kw 3493

#7 MeSH descriptor: [Cerebrovascular Circulation] this term only 1616

#8 (Cerebrovascular Circulation):ti,ab,kw OR (Cerebral Blood Flow):ti,ab,kw OR (CBF):ti,ab,kw OR (arterial spin labeling):ti,ab,kw OR (ASL):ti,ab,kw 5440

#9 MeSH descriptor: [Positron-Emission Tomography] this term only 1061

#10 (Positron Emission Tomography):ti,ab,kw OR (PET):ti,ab,kw 8659

#11 #4or#5or#6or#7or#8or#9or#10 23905

#12 MeSH descriptor: [Cognitive Dysfunction] this term only 1940

#13 (Cognitive Dysfunction):ti,ab,kw OR (Cognitive Decline):ti,ab,kw OR (Mild Cognitive Impairment):ti,ab,kw OR (Cognitive Impairment):ti,ab,kw OR (Cognitive Disorder):ti,ab,kw 33969

#14 (MCI):ti,ab,kw 2808

#15 #12or#13or#14 34704

#16 #3and#11and#15 with Publication Year from 2007 to 2021, in Trials 41

A total of 41 records

Elsevier ScienceDirect

(((Diabetes Mellitus, Type 2[Title/Abstract]) OR (T2DM[Title/Abstract])) AND (((((functional magnetic resonance imaging[Title/Abstract]) OR (functional neuroimaging[Title/Abstract])) OR (positron emission tomography[Title/Abstract])) OR (resting state[Title/Abstract])) OR (cerebral blood flow[Title/Abstract]))) AND ((cognitive dysfunction[Title/Abstract]) OR (mild cognitive impairment[Title/Abstract]))

Review articles (170)

Research articles (56)

Encyclopedia (5)

Book chapters (39)

Conference abstracts (56)

Conference info (1)

Correspondence (1)

Discussion (1)

Editorials (1)

Errata (1)

Mini reviews (5)

Practice guidelines (12)

Short communications (1)

Other (38)

A total of 387 records

Ovid Medline

1 Diabetes Mellitus, Type 2/ or Diabetes Mellitus, Type 2.mp. (150506)

2 limit 1 to yr="2007 -Current" (107503)

3 Type 2 Diabetes.mp. (144845)

4 limit 3 to yr="2007 -Current" (123708)

5 Diabetes Mellitus, Type II.mp.(629)

6 limit 5 to yr="2007 -Current" (371)

7 T2DM.mp. (25685)

8 limit 7 to yr="2007 -Current" (25106)

9 NIDDM.mp.(6967)

10 limit 9 to yr="2007 -Current" (343)

11 2 or 4 or 6 or 8 or 10 (155996)

12 functional neuroimaging.mp. or Functional Neuroimaging/ (9632)

13 limit 12 to yr="2007 -Current" (8108)

14 Functional Brain Imaging.mp. (1748)

15 limit 14 to yr="2007 -Current" (1085)

16 functional magnetic resonance imaging.mp. (34437)

17 limit 16 to yr="2007 -Current" (28823)

18 fMRI.mp. (50561)

19 limit 18 to yr="2007 -Current" (43465)

20 resting state.mp. (24303)

21 limit 20 to yr="2007 -Current" (21673)

22 default network.mp. (561)

23 limit 22 to yr="2007 -Current" (558)

24 ALFF.mp. (853)

25 limit 24 to yr="2007 -Current" (853)

26 ReHo.mp. (795)

27 limit 26 to yr="2007 -Current" (790)

28 Cerebrovascular Circulation.mp. or Cerebrovascular Circulation/ (57285)

29 limit 28 to yr="2007 -Current" (20301)

30 Cerebral Blood Flow.mp. (33431)

31 limit 30 to yr="2007 -Current" (13977)

32 CBF.mp. (14923)

33 limit 32 to yr="2007 -Current" (7104)

34 arterial spin labeling.mp. (3207)

35 limit 34 to yr="2007 -Current" (2948)

36 ASL.mp. (5305)

37 limit 36 to yr="2007 -Current" (4385)

38 Positron Emission Tomography.mp. or Positron-Emission Tomography/ (103816)

39 limit 38 to yr="2007 -Current" (83312)

40 PET.mp.(113096)

41 limit 40 to yr="2007 -Current" (87376)

42 13 or 15 or 17 or 19 or 21 or 23 or 25 or 27 or 29 or 31 or 33 or 35 or 37 or 39 or 41 (204263)

43 Cognitive Dysfunction.mp. or Cognitive Dysfunction/ (40027)

44 limit 43 to yr="2007 -Current" (37244)

45 Cognitive Impairment.mp. (68547)

46 limit 45 to yr="2007 -Current" (58346)

47 Cognitive Decline.mp. (26744)

48 limit 47 to yr="2007 -Current" (23374)

49 Mild Cognitive Impairment.mp. (20307)

50 limit 49 to yr="2007 -Current" (18860)

51 Cognitive Disorder.mp. (947)

52 limit 51 to yr="2007 -Current" (762)

53 MCI.mp. (20087)

54 limit 53 to yr="2007 -Current" (14760)

55 44 or 46 or 48 or 50 or 52 or 54 (93057)

56 11 and 42 and 55 (152)

Ovid MEDLINE(R) ALL <2007 to November 15, 2021>

A total of 152 records

In total, a total of 1223 records were retrieved

frontiers

Table S2. Checklist for objective assessment of methodological quality of studies for neuroimaging meta-analysis

**Category 1: Sample characteristics (10)**
1. Patients were evaluated with specific standardised diagnostic criteria (1)
2. Important demographic data (age and gender) were reported with mean (or median) and standard deviations (or range)) (2)
3. Healthy comparison subjects were evaluated to exclude psychiatric and medical illnesses and demographic data was reported (1)
4. Important clinical variables (e.g. illness duration, onset time, medication status, HAMD scores) were reported with mean (or median) and standard deviations (or range)) (4)
5. Sample size per group > 10 (2)
**Category 2: Methodology and reporting (10)**
1. Whole brain analysis was automated with no a-priori regional selection (3)
2. Magnet strength at least 1.5T (1)
3. At least 5 minutes of resting state acquisition (1)
4. Whole brain coverage of resting scans (1)
5. The acquisition and preprocessing techniques were clearly described so that they could be reproduced (1)
6. Coordinates reported in a standard space (1)
7. Significant results are reported after correction for multiple testing using a standard statistical procedure (FDR, FWE or permutation-based methods) (1)
8. Conclusions were consistent with the results obtained and the limitations were discussed (1)

A maximum score of 20 for each study, allocated as per the criteria specified above.

study	JBI	study	JBI
Xia et al. (2013)	13	Cui et al. (2017)	12
Wang et al. (2014)	13	Dai et al. (2017)	13
Cui et al. (2014)	15	Peng et al. (2017)	12
Xia[a] et al. (2015)	12	Liu et al. (2018)	12
Liu[a] et al. (2015)	13	Yu et al. (2019)	16
Cui et al. (2015)	12	Xia et al. (2020)	13
Xia[b] et al. (2015)	12	Liu[a] et al. (2020)	13
Liu[b] et al. (2015)	12	Li et al. (2020)	12
Cui et al. (2016)	13	Liu[b] et al. (2020)	12
Liu et al. (2016)	12	Xiong et al. (2020)	12
Peng et al. (2016)	14	Feng et al. (2021)	14
Qu et al. (2016)	13		

JBI score greater than 70% of the total score was considered as low risk

Study	Sample characteristics (10)						Methodology and reporting (10)							Quality scores (out of 20)
	Standardised diagnostic criteria	Important demographic data were reported with mean and standard deviations	Exclusion criteria and reporting demogra-phics	Important clinical variables were reported with mean and standard deviations	Sample size per group > 10	Whole brain analysis was automated	Magnet strength at least 1.5T	At least 5 minutes of resting state acquisition	Whole brain coverage of resting scans	The acquisition and preprocessing techniques were clearly described	Coordinates reported in a standard space	Multiple comparison correction	consistent and limitation	
Xia et al. (2013)	☆	☆☆	☆	☆☆☆☆	☆☆	☆☆	☆	☆	☆	☆	☆	☆	☆	19
Wang et al. (2014)	☆	☆☆	☆	☆☆☆	☆☆	☆☆☆	☆	☆		☆	☆	☆	☆	18
Cui et al. (2014)	☆	☆☆	☆	☆☆☆	☆☆	☆☆	☆	☆	☆	☆	☆	☆		17
Xia[a] et al. (2015)	☆	☆☆	☆	☆☆☆☆	☆☆	☆☆☆	☆		☆	☆	☆	☆	☆	19
Liu[a] et al. (2015)	☆	☆☆	☆	☆☆☆	☆☆	☆	☆	☆	☆	☆	☆	☆	☆	17
Cui et al. (2015)	☆	☆☆	☆	☆☆☆☆	☆☆	☆☆☆	☆	☆	☆	☆	☆	☆	☆	20
Xia[b] et al. (2015)	☆	☆☆	☆	☆☆☆	☆☆	☆☆	☆	☆	☆	☆	☆	☆	☆	18
Liu[b] et al. (2015)	☆	☆☆	☆	☆☆☆	☆☆	☆	☆	☆	☆	☆	☆	☆	☆	17
Cui et al. (2016)	☆	☆☆	☆	☆☆☆	☆☆	☆	☆	☆	☆	☆	☆	☆	☆	17
Liu et al. (2016)	☆	☆☆	☆	☆☆☆☆	☆☆	☆☆	☆	☆	☆	☆	☆	☆	☆	19
Peng et al. (2016)	☆	☆☆	☆	☆☆☆☆	☆☆	☆☆☆	☆	☆	☆	☆	☆	☆	☆	20
Qu et al. (2016)	☆	☆☆	☆	☆☆	☆☆	☆☆☆	☆	☆	☆	☆	☆	☆		17
Cui et al. (2017)	☆	☆☆	☆	☆☆☆	☆	☆☆☆	☆		☆	☆	☆	☆	☆	18
Dai et al. (2017)	☆	☆☆	☆	☆☆☆☆	☆	☆☆	☆		☆	☆	☆		☆	17
Peng et al. (2017)	☆	☆☆	☆	☆☆☆	☆☆	☆☆	☆	☆	☆	☆	☆	☆		17
Liu et al. (2018)	☆	☆☆	☆	☆☆☆☆	☆☆	☆☆	☆	☆	☆	☆	☆	☆	☆	19
Yu et al. (2019)	☆	☆☆	☆	☆☆☆☆	☆☆	☆☆☆	☆	☆	☆	☆	☆	☆	☆	20
Xia et al. (2020)	☆	☆☆	☆	☆☆☆	☆☆	☆☆	☆	☆	☆	☆	☆	☆		17
Liu[a] et al. (2020)	☆	☆☆	☆	☆☆☆	☆☆	☆☆	☆	☆	☆	☆	☆	☆	☆	18
Li et al. (2020)	☆	☆☆	☆	☆☆	☆☆	☆☆	☆	☆	☆	☆	☆	☆	☆	17
Liu[b] et al. (2020)	☆	☆☆		☆☆☆	☆☆	☆☆	☆	☆	☆	☆	☆	☆	☆	17
Xiong et al. (2020)	☆	☆☆	☆	☆☆☆	☆☆	☆☆	☆	☆	☆	☆	☆	☆	☆	18
Feng et al. (2021)	☆	☆☆	☆	☆☆☆☆	☆☆	☆☆	☆	☆	☆	☆	☆	☆	☆	18

FigureS1. objective assessment of quality of 23 studies

Table S3. Meta-analytic results of the group one and differences between subgroups

	ICA/DC	Group one		ReHo		
Decrease						
brain label	**R PCG (ROL)**	**R PCG (ROL)**	L LG	undefined	**R PCG (ROL)**	L PCG
MNI coordinate	62, -16, 22	62, -14, 22	-10, -64, 2	-8, -68, 6	58, 2, 10	-56, -20, 24
Number of Voxels	589	1935	410	979	433	75
SDM-Z value	-1.972	-2.762	-2.266	-2.588	-2.427	-2.110
Increase						
brain label	L ACC/PCC	L SFG	R PCu	L SFG	R PCu	
MNI coordinate	-2, 42, 14	-18, 32, 48	18, -60, 36	-18, 30, 50	12, -68, 38	
Number of Voxels	500	1608	402	391	93	
SDM-Z value	1.638	2.345	1.865	2.533	1.682	

Key words: R, right; L, left; PCG, postcentral gyrus; ROL, rolandic operculum; LG, lingual gyrus; ACC/PCC, anterior cingulate / paracingulate gyri; SFG, superior frontal gyrus; PCu, precuneus. Shown in blue font: the results are consistent between the group one and subgroups.

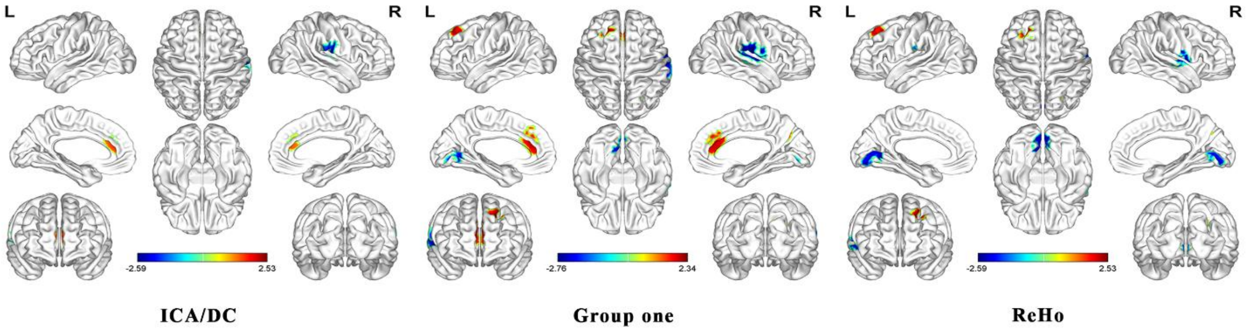

FigureS2. Similarities and differences between the group one analysis and the subgroups analysis.

Table S4. Meta-analytic results of the group two and results of subgroup analysis.

	ALFF	Group two	
Decrease			
brain label	**R LG**	**R LG**	R MTG
MNI coordinate	18, -92, -10	18, -92, -8	44, -74, 28
Number of Voxels	573	272	108
SDM-Z value	-2.702	-2.607	-2.110
Increase			
brain label	**R ACC/PCC**	**R ACC/PCC**; L ACC/PCC	
MNI coordinate	4, 16, 26	8, 12, 34; -6, 12, 38	
Number of Voxels	853	824	
SDM-Z value	2.103	2.057	

Key words: R, right; L, left; LG, lingual gyrus; ACC/PCC, anterior cingulate / paracingulate gyri; MTG, Middle Temporal Gyrus. Shown in blue font: the results are consistent between the group one and subgroups.

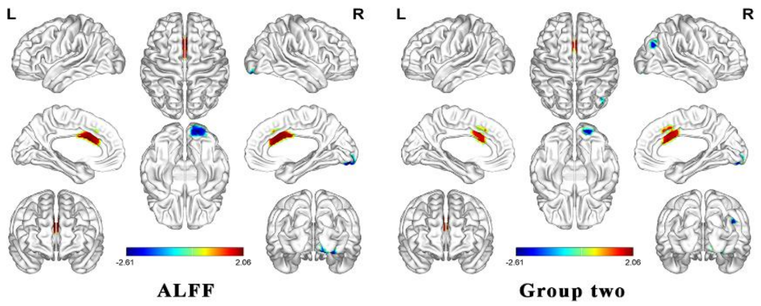

FigureS3. Similarities and differences between the group two analysis and the subgroup analysis

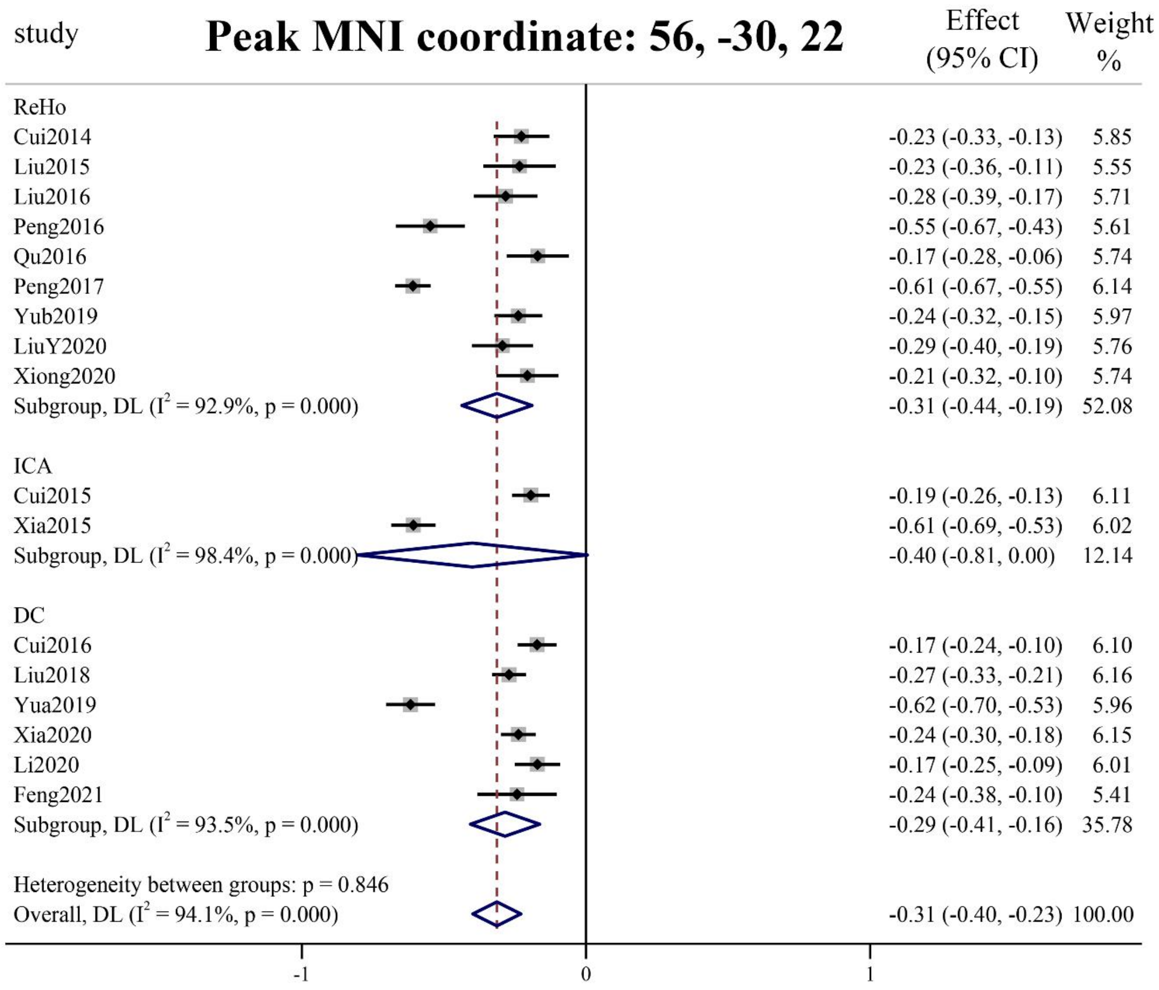

FigureS4. Forest plot of meta-analysis for effect size of right rolandic operculum

TableS5. Egger's test

Std_Eff	Coef.	Std. Err.	t	P>|t|	[95% Conf. Interval]
slope	-.3544467	.1854063	-1.91	0.077	-.7521037, .0432102
bias	.7410512	4.461754	0.17	0.870	-8.828459, 10.31056

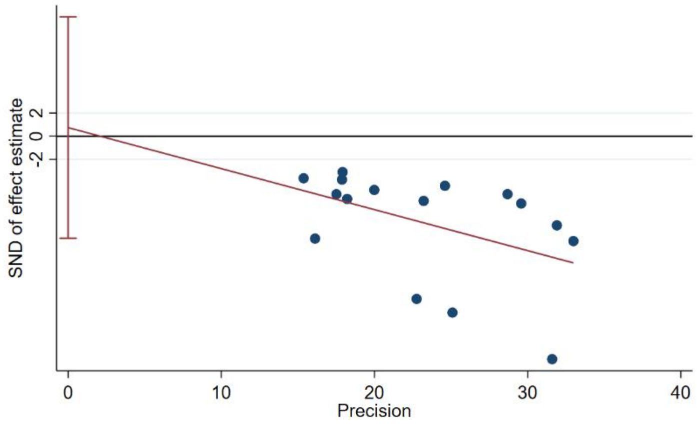

FigureS5. Egger's test
